# Ultrasound Examination of the Penile Urethra in Healthy Non‐Castrated Texel Lambs Confined for Fattening

**DOI:** 10.1111/vru.70244

**Published:** 2026-10-12

**Authors:** Marina Franco, Lisandra de Camargo Campos, Mayara Cardoso dos Anjos, Nicoly Nayana Marcom, Emelly Selmer Moura, Ariadne Solângela Stapait, Priscilla Fajardo Valente Pereira, Júlio Augusto Naylor Lisbôa

**Affiliations:** ^1^ Department of Veterinary Clinics, Universidade Estadual de Londrina Campus Universitário Londrina Paraná Brazil

**Keywords:** reference values, sheep, ultrasonography, urethral lumen width

## Abstract

This study aimed to determine, using ultrasonography, the width of the penile urethral lumen in Texel lambs and to compare two types of linear probes. Thirty healthy, uncastrated lambs kept in confinement for fattening were subjected to ultrasound examinations at 60, 120, and 150 days of age. The penile urethra was examined at four sites: proximal urethra, sigmoid flexure, distal to the sigmoid flexure, and distal urethra. The images were obtained in B‐mode with cross sections using two types of linear probes: for external use (external probe: EP; 13 MHz) and for transrectal use in large animals (rectal probe: RP; 10 MHz). Data were analyzed using a two‐way repeated‐measures ANOVA. The image quality was superior when using EP. The urethra was identified as a circular or elliptical structure with a slightly hyperechoic wall and an anechoic lumen. At the four anatomical sites, the width of the urethra increased with age (*p* < 0.001), and the values measured using RP were higher (*p* < 0.05). With EP, the lumen width was 1.0‒3.4 mm in the proximal urethra, 0.6‒3.3 mm in the sigmoid flexure, 0.5‒2.9 mm in the region distal to the sigmoid flexure, and 0.7‒2.5 mm in the distal urethra; with RP, these values were 0.8‒3.9, 0.6‒3.3, 0.7‒3.3, and 0.8‒2.9 mm, respectively. Ultrasound examination of the penile urethra is feasible, and the values obtained for the lumen width can be accepted as physiological standards for comparison. This examination can be performed with a linear RP in large animals.

## 1 Introduction

Urolithiasis is a common disease in sheep, especially in males. It is associated with the formation of uroliths that can obstruct the urinary tract, making it difficult to eliminate urine and causing complications that can lead to death [1, 2]. This disease is characterized by the formation of stones composed of a combination of minerals, such as calcium, phosphorus, and magnesium, and organic substances, such as ammonia and oxalate [3, 4, 5]. In sheep, obstructive urolithiasis is most often associated with the ingestion of concentrated diets with excess phosphorus and insufficient water intake, which favors solute precipitation [2, 4, 6]. Another important factor is the castration of very young lambs, which compromises the normal development of the urinary tract, resulting in a narrower urethra and an increased risk of obstruction [1]. The frequency of this disease was reported as 5.1% in sheep and goats in a Brazilian study, in which sheep represented 83.7% of all cases [4].

Urinary flow obstruction can lead to bladder distension, severe pain, and urinary infections; if not treated properly, it can result in kidney failure, rupture of the bladder or urethra, and death [7, 8]. Early diagnosis and the adoption of immediate therapeutic measures are crucial for reducing mortality [1, 2]. The diagnosis of obstructive urolithiasis in sheep is based on clinical evaluation, including the identification of signs such as abdominal pain and dysuria [9, 10], as well as radiographic [7, 11] and ultrasonographic [12, 13, 14] examinations; the latter method has greater applicability in practice under field conditions.

The changes observed on ultrasonography include a distended urinary bladder and hydronephrosis, represented by dilation of the renal pelvis and increased kidney volume, with dilation of the ureter and uroliths in the urinary bladder being more difficult to detect [6, 10, 13, 14, 15, 16]. Dilation of the pelvic urethra in its initial portion close to the bladder can be visualized through transrectal assessment [15]. A dilated penile urethra can be observed; however, it is generally considered a difficult structure to visualize [15, 16].

Ultrasound examination of the penile urethra of lambs is possible by positioning the linear probe for external use in the perineal region, at the base of the scrotum, and in the preputial region [17]. In this study, the widths of the penile urethral lumen in healthy crossed Lacaune lambs aged 3 weeks and 4 months were measured. The authors of the present study are unaware of other information on the subject available in the literature. Knowledge of the dimensions of the penile urethral lumen in healthy lambs is relevant, as it serves as a confirmatory comparison parameter in cases of urethral dilation due to the accumulation of urine.

This study aimed to determine, using ultrasonography, the width of the penile urethral lumen in clinically healthy, uncastrated Texel lambs confined for fattening between 2 and 5 months of age and to compare two types of linear ultrasonography probes for this purpose.

## 2 Materials and Methods

This observational study with a repeated‐measures design was previously approved by the Ethics Committee on the Use of Animals of the Universidade Estadual de Londrina, under protocol number CEUA/UEL 022.2022.

### 2.1 Animals and Management

The study included 30 healthy Texel lambs (uncastrated males between 2 and 5 months of age), weaned and kept confined for fattening. The animals were weaned at 2 months of age and kept in a confinement system for fattening until 5 months of age.

The diet consisted of 85% concentrated foods and 15% corn silage. The following components were used for the concentrated portion: corn grain, soybean meal, and a commercial mineral core, making up 16% crude protein and 75% total digestible nutrients. Water was provided ad libitum. Before being weaned and confined, the lambs had free access to the same diet for voluntary intake in a creep‐feeding system.

Confinement was carried out in a community shed with sufficient area to house up to 200 lambs, which meant an area of at least 4 m^2^/head. The floor was made of unpaved soil covered with wood shavings in the areas close to the troughs. Six feeding troughs were distributed throughout the shed with an approximate trough linear area of 30 cm/head. Three water troughs were used. Cleaning of the troughs and floor of the shed, involving the removal of feces and food waste and changing the shavings’ bedding, was carried out once a week.

The confined lambs were observed daily by the farm's keepers.

### 2.2 Procedures

The lambs selected for the study were weighed using a conventional scale at 2 and 5 months of age. Before each ultrasound examination, they underwent routine physical examination with measurement of rectal temperature, heart rate, respiratory rate, and rumen motility, and assessment of behavior, mucous membrane color, hydration status, and fecal characteristics. Only the lambs that remained clinically healthy throughout the monitoring period participated in the study.

Ultrasound examination was performed at the following ages: 60 (±5) days (beginning of the confinement period), 120 (±5) days, and 150 (±5) days (end of the confinement period). The lambs were physically restrained and maintained in right lateral recumbency. The areas defined for capturing the images were prepared with trichotomy using a portable hair trimmer. An ultrasound‐conductive gel was applied between the skin and the probe surface to increase the contact surface between the probe and skin, maintaining the best possible image quality.

The penile urethra was systematically examined at four specific locations (Figure 1): (a) proximal urethra (perineal region), (b) sigmoid flexure (caudal aspect of the base of the scrotum), (c) distal to the sigmoid flexure (cranial aspect of the base of the scrotum), and (d) distal urethra (halfway along the distance between the glans and the base of the scrotum). The images were captured with cross sections. At each location, the luminal diameter was measured when the urethra was circular. The long axis of the lumen was measured when it was elliptical. In exceptional cases where the urethra was dilated with urine, measurements were taken after urination was induced by brief apnea.

**FIGURE 1:**
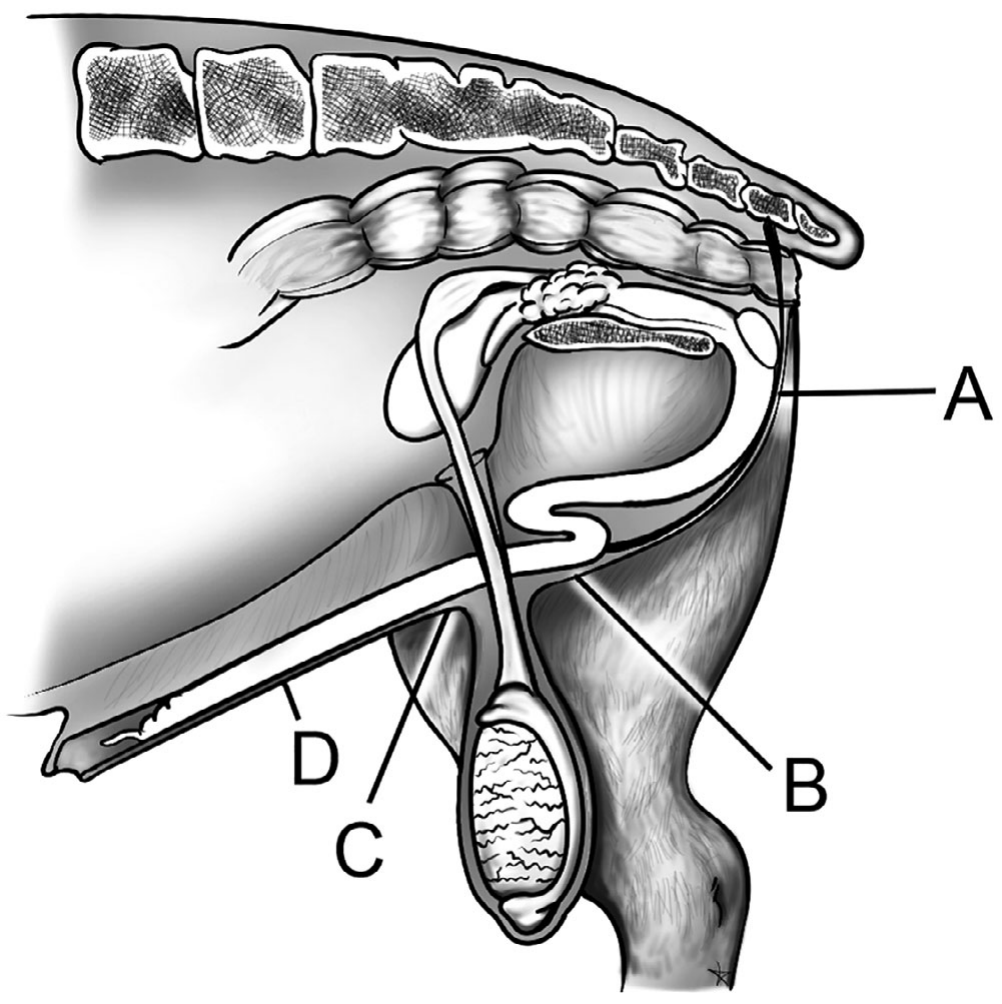
Anatomical locations of the penile urethra evaluated by ultrasonography in healthy Texel lambs (between 2 and 5 months of age) confined for fattening. A—proximal urethra; B—sigmoid flexure; C—distal to the sigmoid flexure; and D—distal urethra.

An ultrasonography device (MyLab Delta, Esaote Veterinary, Genova, Italy) was used. The evaluations were performed in B‐mode using two types of probes: a linear probe for external use (external probe: EP; model SL 1543) and a linear probe for transrectal use in large animals (rectal probe: RP; model SV 3513), with the highest possible frequency for each probe (13 and 10 MHz, respectively).

### 2.3 Statistical Analysis

Two‐way repeated‐measures ANOVA was used to test the effect of age (2 × 4 × 5 months), the effect of the type of linear probe used (EP × RP), and the interaction between these two factors. Each urethral anatomical site was considered separately in this analysis. One‐way ANOVA was used to compare the width of the urethral lumen at the four anatomical locations examined using the EP. For this analysis, readings of the three ages were considered for each anatomical location for a total of 90 measurements. Tukey's test was used for multiple comparisons, assuming a probability of error of 5%. The analyses were performed using SigmaPlot 13.0 (Systat Software Inc., San Jose, CA, USA) software.

## 3 Results

The lambs weighed 15.7 ± 3.4 kg at 2 months of age and 37.1 ± 4.3 kg at 5 months of age.

Table 1 presents the results of the study. At the four anatomical sites examined, the width of the urethra increased with advancing age (*p* < 0.001). The effect of the type of probe used was confirmed in the proximal urethra (*p* = 0.048), in the sigmoid flexure (*p* = 0.011), in the region distal to the sigmoid flexure (*p* = 0.003), and in the distal urethra (*p* < 0.001); the values measured using RP were higher. The interaction between age and type of probe used was not significant at any of the sites examined (*p* = 0.070, 0.992, 0.304, and 0.783, respectively). Nevertheless, we chose to compare the probes at each age (Table 1).

**TABLE 1:** Age variation (mean ± standard deviation) of the width of the penile urethral lumen (mm) of Texel lambs kept confined for fattening, measured in four anatomical locations using a conventional linear probe for external use (external probe [EP]; 13 MHz) and a linear probe for transrectal use in large animals (rectal probe [RP]; 10 MHz).

Anatomical site	Probe	2 months	4 months	5 months
				
Proximal urethra^1^	EP	1.91 ± 0.44^Ab^	2.03 ± 0.66^Aab^	2.27 ± 0.57^Ba^
	RP	1.76 ± 0.45^Ab^	2.22 ± 0.63^Aa^	2.54 ± 0.69^Aa^
				
Sigmoid flexure^2^	EP	1.42 ± 0.40^Ac^	1.75 ± 0.43^Ab^	2.11 ± 0.51^Aa^
	RP	1.58 ± 0.48^Ab^	1.93 ± 0.51^Aa^	2.29 ± 0.53^Aa^
				
Distal to the sigmoid flexure^3^	EP	1.42 ± 0.45^Ab^	1.54 ± 0.42^Bb^	1.98 ± 0.47^Ba^
	RP	1.52 ± 0.39^Ab^	1.82 ± 0.45^Ab^	2.16 ± 0.57^Aa^
				
Distal urethra^4^	EP	1.47 ± 0.38^Ab^	1.67 ± 0.38^Bb^	1.95 ± 0.29^Ba^
	RP	1.64 ± 0.42^Ab^	1.94 ± 0.49^Aa^	2.14 ± 0.40^Aa^

*Note*: Different capital letters (A and B) represent differences between probes (*p* < 0.05). Different lowercase letters (a–c) represent differences between ages (*p* < 0.05).

^1^ perineum 2‒3 cm distal to the anus.

^2^ caudal to the base of the scrotum.

^3^ cranial to the base of the scrotum.

^4^ half the distance between the cranial aspect of the base of the scrotum and the glans.

Considering the total number of measurements taken at each anatomical site at the three ages using EP, the width of the lumen of the proximal urethra (2.07 ± 0.58 mm) was greater (*p* < 0.001) than those observed at the sigmoid flexure (1.74 ± 0.52 mm), distal to the sigmoid flexure (1.67 ± 0.51 mm), and at the distal urethra (1.71 ± 0.40 mm), which did not differ from each other.

Failures in visualizing the urethra occurred using EP (10/90; 11.1%) but were more frequent using RP (22/90; 24.4%). In examinations performed with EP, failures were more frequent in the sigmoid flexure (5/90; 5.5%) and distal to the sigmoid flexure (4/90; 4.4%). In examinations performed using RP, failures were more frequent in the sigmoid flexure (12/90; 13.3%) and distal to the sigmoid flexure (6/90; 6.6%). With both probes, failures were more frequent at 2 months of age (6/30 [20%] with EP and 10/30 [33.3%] with RP).

The quality of the images obtained was always higher using EP than using RP. The urethra was identified as a circular or elliptical structure with a slightly hyperechoic wall and an anechoic lumen (Figures 2, 3, 4, 5). In the images of the proximal penile urethra, the body of the penis was hardly visualized with defined edges (Figure 2). Visualization of the body of the penis was always possible at the other examined locations (Figures 3, 4, 5). In most cases, the hyperechoic wall of the penile urethra at the sigmoid flexure and distal to the sigmoid flexure was not completely visualized (Figures 3 and 4 ). It was possible to observe only the ventral (Figure 3) or dorsal (Figure 4) wall, regardless of the location examined. In the perineal region, obtaining the image of the proximal penile urethra could be compromised with the use of RP owing to the impossibility of perfect coaptation of the probe's acoustic lens with the surface of the skin due to the large semimembranous muscles in the breed studied. This difficulty was observed from the age of 2 months and became more pronounced with advancing age.

**FIGURE 2:**
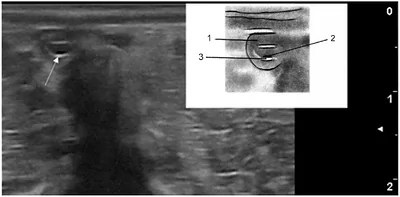
Ultrasonographic image of the proximal penile urethra (arrow) of a 2‐month‐old uncastrated Texel lamb kept confined for fattening. Cross‐section is in the perineal region (2‒3 cm below the anus), using the linear probe for external use (13 MHz). 1—body of the penis; 2—urethral lumen; 3—urethral wall.

**FIGURE 3:**
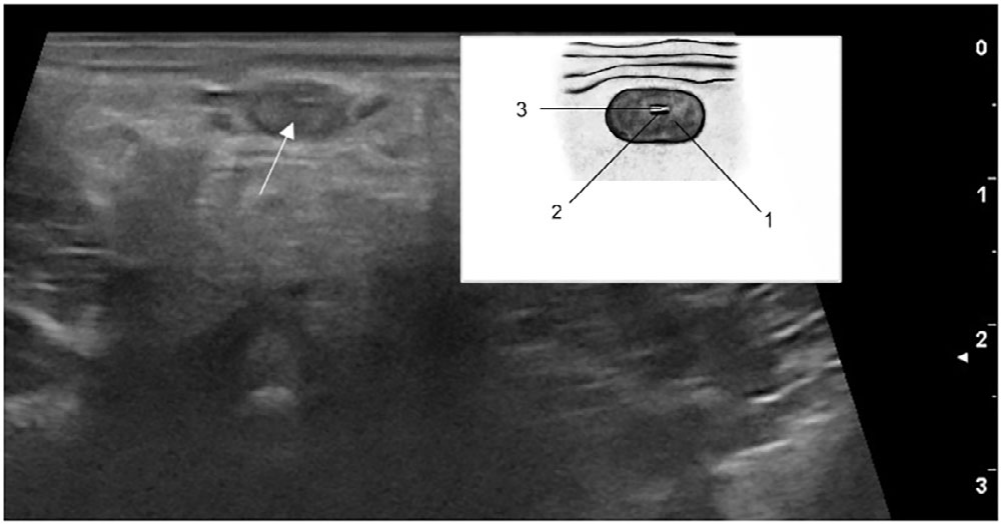
Ultrasonographic image of the penile urethra (arrow) in the sigmoid flexure of a 2‐month‐old uncastrated Texel lamb kept confined for fattening. Cross‐section is on the caudal aspect of the base of the scrotum, using the linear probe for external use (13 MHz). 1—body of the penis; 2—urethral lumen; 3—urethral wall.

**FIGURE 4:**
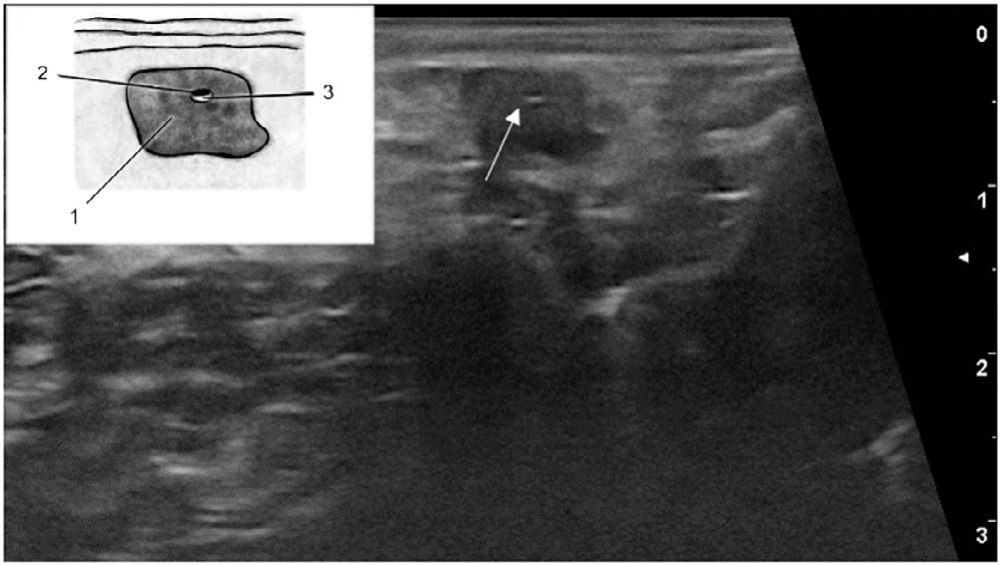
Ultrasonographic image of the penile urethra (arrow) distal to the sigmoid flexure of a 2‐month‐old uncastrated Texel lamb kept confined for fattening. Cross‐section is on the cranial aspect of the base of the scrotum, using the linear probe for external use (13 MHz). 1—body of the penis; 2—urethral lumen; 3—urethral wall.

**FIGURE 5:**
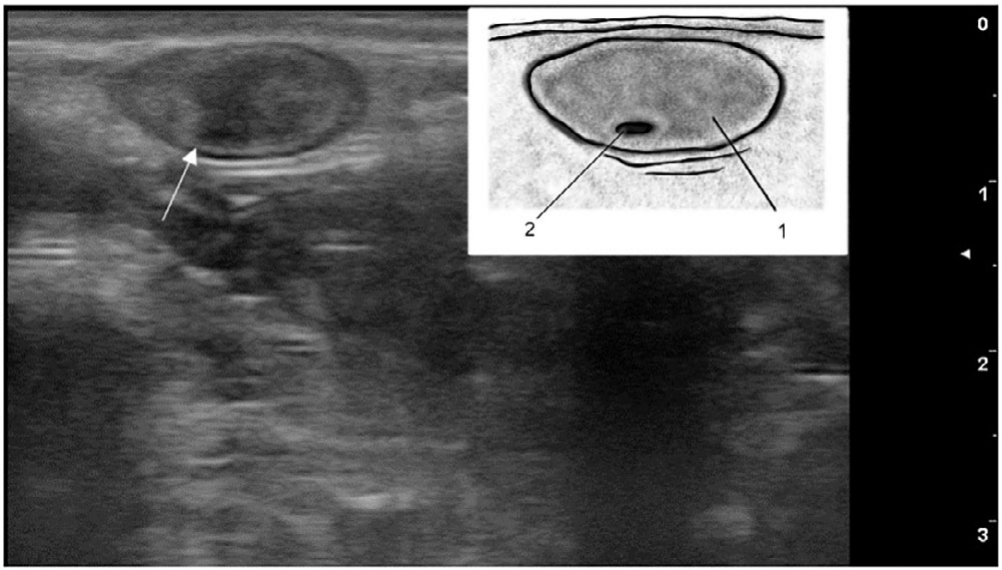
Ultrasonographic image of the distal penile urethra (arrow) of a 4‐month‐old uncastrated Texel lamb kept confined for fattening. Cross‐section is at half the distance between the cranial aspect of the base of the scrotum and the glans, using the linear probe for external use (13 MHz). 1—body of the penis; 2—urethral lumen.

Considering the four anatomical locations examined, an elliptical shape was predominant using EP (269/350; 76.8%) as well as RP (273/338; 80.7%). The distal urethra stood out as the site with the highest frequency of a circular shape: 46.0% (41/89) using EP and 33.7% (30/89) using RP.

## 4 Discussion

The results of this study are relevant because they can be used to diagnose obstructive urolithiasis in lambs. The width of the penile urethral lumen observed in healthy lambs can serve as an objective comparison parameter, confirming the presence of urethral dilation with retained urine in cases of obstruction of this structure. In lambs affected by this disease, the flow of urine is reduced or impeded, most commonly owing to the accommodation of uroliths in the urethra [10, 13], as the formation of these urinary stones occurs more frequently in the urinary bladder than in the kidneys [2, 7].

According to Farver [18], reference values must be established by considering the interval between two standard deviations below and above the mean. With the use of EP, the width of the lumen of the penile urethra showed variation, being 1.0‒3.4 mm in the proximal urethra, 0.6‒3.3 mm in the sigmoid flexure, 0.5‒2.9 mm in the region distal to the sigmoid flexure, and 0.7‒2.5 mm in the distal urethra; with RP, these values were 0.8‒3.9, 0.6‒3.3, 0.7‒3.3, and 0.8‒2.9 mm, respectively. It can be assumed, therefore, that in Texel lambs aged 2‒5 months, the width of the penile urethral lumen being greater than 3.5 mm with the use of EP and greater than 4.0 mm with the use of RP is indicative of urethral dilation with retained urine.

During the monitoring period in the herd studied, there were no cases of obstructive urolithiasis in the lambs confined for fattening. Therefore, it was not possible to identify the dilation of the penile urethra and compare it with the lumen width values observed under physiological conditions. However, in rare cases, the penile urethra has been visualized dilated with retained urine preceding urination. Although increased widths were not measured in these cases, it is important to note that the penile urethra, which is dilated with urine, is much more easily identified, standing out as a circular structure with an evident anechoic lumen. It is reasonable to assume that the same ease of visualization occurs in cases of obstructive urolithiasis, which makes ultrasound examination efficient in confirming the diagnosis of this disease.

The values obtained for the width of the urethral lumen in the studied lambs were compatible with those observed in a previous study, wherein the pelvic urethra was examined by transrectal ultrasonography in its initial portion close to the urinary bladder, with a diameter of 1‒3 mm for the internal orifice in the bladder of healthy adult sheep [15]. However, unlike in the present study, attempts to examine the penile urethra were unsuccessful, both in transverse and longitudinal sections, using a 5 MHz convex probe positioned externally. The impossibility of obtaining defined images of the penile urethra may have occurred for two reasons: the use of a probe with a low frequency, and the reduced quality of definition of the images generated in older ultrasonography devices. Indeed, 32 years separate the two studies, and the technological advancement of ultrasonography equipment is undeniable, which may justify the discrepancy observed between the results of the studies.

Compared to the results observed in the unique previous study wherein ultrasonographic evaluations of the penile urethra of lambs were carried out [17], the average values of urethral lumen width measured in the present study are three to four times greater. These authors found that the average diameter of the urethra varied from 0.47 to 0.54 mm in healthy uncastrated Lacaune cross lambs aged 4 months. A direct comparison between the two studies is justified because both used linear probes for external use with similar frequencies (12 and 13 MHz) and were positioned in anatomical locations of the urethra that were also relatively similar. The discrepancy between the results of the two studies can probably be explained by differences between the breeds examined.

The width of the lumen of the penile urethra increased with advancing age in the lambs studied, generally assuming higher values at 5 months than those observed at 2 months of age. The biological relevance of this difference is questionable owing to the small magnitude of the increase in values. The observed age variation contrasts with the results reported in Lacaune cross lambs, which showed no difference between the ages of 3 weeks and 4 months, despite having tripled their body weight [17]. The small increase in the width of the urethral lumen in the lambs studied can be explained by the small difference between the ages evaluated. In the present study, the ages defined for ultrasound evaluation were those representing the beginning (2 months) and end (5 months) of the confinement period for fattening. This decision was based on exposure to an important risk factor for the development of urolithiasis in lambs, as they were maintained on a diet rich in concentrates [2, 6].

The penile urethra was examined at four predefined anatomical locations according to the logic that urethral dilation occurs proximal to the site of the obstruction. Therefore, even if uroliths are not visualized on ultrasonography, urethral dilation with retained urine allows the obstruction site to be defined. Considering that the two critical sites for obstruction with uroliths in lambs are the sigmoid flexure and urethral process [2, 7], it is reasonable to assume that in the first case, the proximal urethra is dilated as opposed to the position distal to the sigmoid flexure and distal urethra. Conversely, in cases of obstruction in the urethral process, the penile urethra is dilated along its entire length. Therefore, it can be assumed that this systematic ultrasound examination of the penile urethra is valuable for the diagnosis of obstructive urolithiasis and can contribute to making the most appropriate therapeutic decisions in each case. These assumptions should be confirmed in future studies.

The results of the present study confirm the previous statement that the linear probe for transrectal use in large animals, when positioned externally, can be used for ultrasound examination of the structures of the urinary tract of small ruminants, including the penile urethra [16]. The images obtained using RP had a lower definition than those obtained using EP, and the width values of the urethral lumen were slightly greater using RP at 4 and 5 months of age, in the region distal to the sigmoid flexure, and in the distal urethra. These differences may be related to the lower frequency of RP (10 MHz) than that of EP (13 MHz). Despite the differences observed between the probes studied, it can be stated that the externally positioned RP is also efficient for ultrasound examination of the penile urethra in lambs. This is relevant because many veterinarians who work with large animals in farm conditions only use RP owing to its routine application, particularly in gynecological examinations. The fact that they do not use EP does not prevent these clinicians from performing ultrasound examination of the penile urethra in lambs.

Finally, it is important to point out as the main limitation of this study the impossibility of examining lambs with obstructive urolithiasis, which prevented comparison with the results observed in a physiological condition.

## 5 Conclusion

Ultrasound examination of the penile urethra of Texel lambs between 2 and 5 months of age is viable, and the values obtained for the width of the lumen can be accepted as a physiological standard for comparison. Penile urethral widths greater than 3.5 mm using EP and greater than 4.0 mm using RP are indicative of urethral dilation with retained urine. This examination can be performed using a linear probe for rectal use in large animals at a frequency of 10 MHz.

## Author Contributions

**Marina Franco**: writing – review and editing. **Lisandra de Camargo Campos**: writing – original draft. **Mayara Cardoso dos Anjos**: software. **Nicoly Nayana Marcom**: validation. **Emelly Selmer Moura**: methodology. **Ariadne Solângela Stapait**: visualization. **Priscilla Fajardo Valente Pereira**: conceptualization. **Júlio Augusto Naylor Lisbôa**: resources.

## Funding

The authors have nothing to report.

## Conflicts of Interest

The authors declare no conflicts of interest.

## Previous Presentation or Publication

We confirm that this work has not been published (except as an academic thesis), and that it is not under consideration for publication elsewhere.

## Checklist

The CONSORT checklist was followed where applicable.

## Data Availability

The data are available from the corresponding author upon reasonable request.
